# “SIT” with *Emx1*‐NuTRAP Mice: Simultaneous INTACT and TRAP for Paired Transcriptomic and Epigenetic Sequencing

**DOI:** 10.1002/cpz1.570

**Published:** 2022-10-26

**Authors:** Anthony M. Raus, Nellie E. Nelson, Tyson D. Fuller, Autumn S. Ivy

**Affiliations:** ^1^ Physiology and Biophysics University of California, Irvine, School of Medicine Irvine California; ^2^ Pediatrics University of California, Irvine, School of Medicine Irvine California; ^3^ Anatomy and Neurobiology University of California, Irvine, School of Medicine Irvine California; ^4^ Division of Neurology Children's Hospital Orange County Orange California

**Keywords:** epigenetics, hippocampus, neurons, transcription

## Abstract

Epigenetic regulation of transcription is gaining increasing importance in the study of neurobiology. The advent of sequencing technology has enabled the study of this regulation across the entire genome and transcriptome. However, modern methods that allow the correlation of transcriptomic data with epigenomic regulation have had several key limitations, including use of separate tissue sources and detection of low‐expression genes. This article describes a method combining isolation of nuclei tagged in specific cell types (INTACT) with translating ribosome affinity purification (TRAP) in the same cell homogenate, referred to as Simultaneous INTACT and TRAP (SIT). We used this technical approach to directly couple transcriptomic sequencing with epigenomic data in neurons derived from the mouse hippocampus. We demonstrate this method with an *Emx1*‐NuTRAP transgenic mouse model. Here, we present protocols for SIT and for the generation and validation of the *Emx1*‐NuTRAP mouse model that we used to demonstrate SIT. These methods enable cell type–specific comparison of translating mRNA and chromatin data from the same set of cells. Using SIT and the *Emx1*‐NuTRAP transgenic mouse model, researchers can compare epigenomic data to transcriptomic data in the same set of hippocampal excitatory neurons. © 2022 The Authors. Current Protocols published by Wiley Periodicals LLC.

**Basic Protocol 1**: *Emx1*‐NuTRAP transgenic mouse line for labeling excitatory neurons in the hippocampus

**Basic Protocol 2**: SIT: Simultaneous Isolation of nuclei tagged in specific cell types (INTACT) and Translating ribosome affinity purification (TRAP)

## INTRODUCTION

Epigenetic processes inform transcriptional regulation within cells, which can in turn inform their function. Methods for correlating the epigenetic landscape and transcriptional output will be paramount to understanding these regulatory networks and how they are modulated by activity or environmental experience. With the generation of the NuTRAP mouse (Roh et al., 2017), it became possible to isolate translating mRNA and nuclei from select cells labeled with a promoter‐specific Cre driver. However, an animal expressing this cassette specifically in hippocampal excitatory neurons had yet to be generated. We study the interaction between epigenetic regulation and transcription in the brain after early life experiences (Raus et al., 2022), so we developed a mouse and a method to investigate these connected regulatory networks simultaneously. By crossing an *Emx1*‐Cre mouse (Gorski et al., 2002) with the NuTRAP mouse (Roh et al., 2017), we generated a transgenic mouse line that labels excitatory neurons in the hippocampus with both nuclear (mCherry and biotin) and ribosomal (GFP) tags. To interrogate the epigenome and transcriptome of excitatory neurons, we developed a method for performing both isolation of nuclei tagged in specific cell types (INTACT) and translating ribosome affinity purification (TRAP) on the same set of cells. We call this method “SIT,” for Simultaneous INTACT and TRAP. Ultimately, the method isolates translating mRNA and nuclei from the same set of cells labeled for cell‐type specificity by the NuTRAP mouse model. Previous methods had been limited by performing TRAP and INTACT on separate tissue samples (Roh et al., 2017). Brain regions are highly heterogeneous, and small differences in cell type within the region could correspond to large differences in function, even among neurons. Our method allows the translatome and epigenome to be studied in concert, from the same set of cells, to allow for better correlation of translating mRNA with epigenetic regulation. The isolated TRAP mRNA and nuclei can be used in downstream molecular analysis applications, such as RNA‐seq, CHIP‐seq, CUT&RUN‐seq, and ATAC‐seq. Any cell type labeled with the NuTRAP cassette could be used with this method. This enables multi‐omic sequencing data to be gathered from the same sample (which has the potential to reduce the number of samples needed in a given study) and helps to overcome population heterogeneity problems associated with traditional bulk sequencing approaches.

Here, we present a protocol for generation of the *Emx1*‐NuTRAP transgenic mouse line for the labeling of excitatory neurons in the hippocampus (Basic Protocol 1) and our “SIT” protocol for the simultaneous isolation of mRNA and nuclei from specific cell types (Basic Protocol 2). We provide sample data to demonstrate the success of our transgenic mouse labeling and the process by which we isolate the mRNA and chromatin.

## STRATEGIC PLANNING

Basic Protocol 2 is labor intensive and has a number of time‐sensitive steps, especially if RNA integrity is a concern, such as for samples frozen for long periods of time. We recommend that at least two people proceed with the protocol until the nuclei are isolated. We recommend that all buffers be prepared the day before [except for the final addition of dithiothreitol (DTT) and cycloheximide]. It takes ∼11 hr to complete this protocol if the experimenter chooses to perform a 2‐hr TRAP incubation. The alternative is to perform this incubation overnight. The shorter method can be used if there is concern for RNA integrity.

Preparation of the protein G Dynabeads with or without GFP may be done 1 day prior to starting Basic Protocol 2, and the beads can be stored overnight at 4°C. The final bead pull‐down and elution should be done with freshly prepared buffers. This preparation reduces the number of preparation steps required on the day of the protocol.

*NOTE*: Conduct experiments and care for the mice according to the US National Institutes of Health Guidelines for Animal Care and Use.

## Basic Protocol 1 *E* *mx1*‐NuTRAP TRANSGENIC MOUSE LINE FOR LABELING EXCITATORY NEURONS IN THE HIPPOCAMPUS

This protocol includes a breeding scheme that generates a line of mice that have excitatory neurons in the neocortex (including the hippocampus) labeled with biotin and mCherry on the nuclear membrane and eGFP on ribosomes. *Emx1*‐NuTRAP mice are healthy, with no signs of phenotypic abnormalities.

### Materials

Female *Emx1*‐IRES‐Cre knock‐in breeder mice (Jackson Laboratory, strain no. 005628)
Male NuTRAP breeder mice (Jackson Laboratory, strain no. 029899)

Additional reagents and equipment for mouse perfusion, brain removal, tissue sectioning, and immunofluorescence imaging (see Current Protocols article: Paletzki & Gerfen, 2019; Tu et al., 2021)

*NOTE*: We recommend ordering at least three female breeders; in our experience, crossing *Emx1*‐Cre females with NuTRAP males yields litter sizes of 4 to 8 pups. Three breeding pairs are usually sufficient to produce enough *Emx1*‐NuTRAP offspring of the same sex to generate four mice/sex/group.

*NOTE*: The *Emx1*‐Cre mouse line contributes the Cre driven by the excitatory cortical neuron–specific promoter (*Emx1* promoter) that activates the NuTRAP cassette in the *Emx1*‐NuTRAP mouse (Gorski et al., 2002). The NuTRAP mouse line contributes the NuTRAP cassette for labeling the cells expressing Cre with biotin and mCherry on their nucleus and eGFP on their ribosomes (Roh et al., 2017).

*NOTE*: A vivarium with proper environmental controls, housing, and animal feed according to the recommendations of the Jackson Laboratory is required.

**Figure 1:**
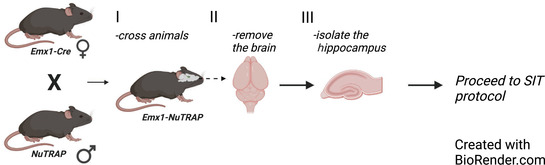
Diagram of Basic Protocol 1 for generation of *Emx1*‐NuTRAP mice and dissection of the hippocampus from those mice.

1 Cross female *Emx1*‐IRES‐Cre knock‐in breeder mice with male NuTRAP breeder mice (see Fig. 1, step I).
  We do not use male Emx1‐IRES‐Cre animals in this cross. Emx1 may allow active Cre in sperm.

**Figure 2:**
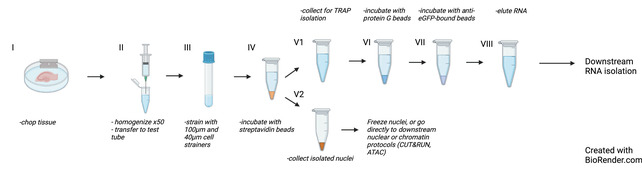
Schematic of the major steps of the SIT protocol (Basic Protocol 2).

2 Optional: Separate males from the dams when a pregnancy is noted.
  We have found that this decreases the litter cannibalization rate.
3 Wean pups from their dams at P21.
  Earlier weaning may be possible, but qualitatively, we observe this to be a weaning time with high pup survival.
4 To confirm that the ribosomes and nuclei are fluorescently labeled specifically in hippocampal neurons, perfuse mice, remove brain, section tissue, and perform immunofluorescence imaging according to Tu et al. (2021) or Current Protocols article by Paletzki & Gerfen (2019).
  We use a traditional immunofluorescent microscope (Keyence BZ‐X800), rather than the scanner described in Tu et al. (2021).
  The mCherry fluorophore could be used to enhance visualization of nuclear labeling.
  The pairing of homozygous Emx1‐Cre with homozygous NuTRAP can only produce offspring that are heterozygous for both alleles. We recommend examining the NuTRAP‐expressing tissue by fluorescence microscopy for one mouse from each new pairing to confirm that the parent mice indeed were the intended mice.

## Basic Protocol 2 SIT: SIMULTANEOUS ISOLATION OF NUCLEI TAGGED IN SPECIFIC CELL TYPES (INTACT) AND TRANSLATING RIBOSOME AFFINITY PURIFICATION (TRAP)

This protocol isolates eGFP‐tagged ribosomes bound to translating mRNA and nuclei from a single homogenate of NuTRAP‐tagged cells. The goal is to pair bulk mRNA sequencing data with epigenetic sequencing data derived from the same set of cells, as this may more directly reflect the epigenetic regulatory mechanisms involved in the observed differential gene expression. The protocol begins with a preparation section that is critical for efficient execution of SIT, and it is important enough that we include it here rather than in the Strategic Planning section. These steps are followed by tissue homogenization and suspension of the cells in a solution containing cycloheximide to bind the mRNA to the ribosomes. The protocol continues with lysing the cell membrane in a buffer followed by filtration with cell strainers that leave the nuclear membrane intact. Then, the biotin‐tagged nuclei are incubated with and bound to streptavidin‐coated beads, and the supernatant containing the cytoplasmic lysate is employed in a modified TRAP protocol. The nuclei from this preparation can be used in many downstream chromatin analysis applications. Similarly, the isolated TRAP mRNA can be applied in many downstream analyses, such as sequencing.

### Materials

1× phosphate‐buffered saline (PBS; 1:10 dilution of 10× PBS in molecular‐grade water; Genesee Scientific, cat. no. 25‐507XB)
dl‐Dithiothreitol (DTT), molecular biology grade (Sigma‐Aldrich, cat. no. D9779‐1G)
Cycloheximide, 98% (Fisher Scientific, cat. no. NC9651091)
Nuclear preparation buffer (NPB; see recipe), without cycloheximide and DTT added
Concentrated TRAP isolation buffer (CTIB; see recipe; make fresh), without cycloheximide and DTT added
High‐salt wash buffer (HSWB; see recipe), without cycloheximide and DTT added
INTACT buffer (IB; see recipe)
Protein G Dynabeads (Novex magnetic beads DYNAL Dynabeads, protein G, Fisher Scientific, cat. no. 10003D)
Mouse anti‐GFP antibody (Sigma, cat. no. G6539‐100UL, lot no. 128M4867V)
*Emx1*‐NuTRAP mice (see Basic Protocol 1; or another transgenic line expressing NuTRAP cassette in select cell type of interest)
Streptavidin‐coated Dynabeads (SCD; Dynabeads M‐280 Streptavidin, Life Technologies, cat. no. 11205D)
RNeasy Mini Kit (QIAGEN, cat. no. 74134), including RLT, RW1, RDD, and RPE buffers; spin columns; 2‐ml collection tubes; and RNase‐free water
70% (v/v) ethanol
RNase‐free DNase (QIAGEN, cat. no. 79254)

1.7‐ml microcentrifuge tubes (Laguna, cat. no. 4060C)
Vortex
Magnetic stand (MagneSphere Technology Magnetic Separation Stand, 12 position, 1.5 ml, Fisher Scientific, cat. no. PRZ5342)
Nutator (MiniMixer 3‐D Shaker Nutator, “10.5 × 7.5” Dimpled Mat, VWR, cat. no. 490012‐328)
Razor blades
Forceps
Dissection scissors
Sterile filter paper–covered petri dishes
Spatulas
18G needles (BD PrecisionGlide^TM^ Needle 18G, BD, cat. no. 305195)
1‐ml syringes (BD, cat. no. 309659)
100‐μm cell strainer caps (yellow) on 5‐ml tubes (Bundle pluriStrainer® Mini 100 µm and Flow Cytometry Tubes, pluriSelect USA, cat. no. 43‐10100‐46)
Benchtop centrifuge (with 5424 R 24 × 2 ml AT‐ROTOR, Eppendorf, cat. no. 5404000537), 4°C
40‐μm cell strainer caps (blue) on 5‐ml tubes (Bundle pluriStrainer^®^ Mini 40 µm and Flow Cytometry Tubes, pluriSelect USA, cat. no. 43‐10040‐46)
Microcentrifuge, 4°C
Mini centrifuge (TSZ mini centrifuge, max speed 7000 rpm, Fisher Scientific, cat. no. 507514951)
Fluorometer [Qubit 4 Fluorometer, University of California, Irvine, Genomics High Throughput Facility (UCIGHTF)]
Bioanalyzer (Agilent Bioanalyzer 2100, UCIGHTF)

Additional reagents and equipment for mouse sacrifice

*CAUTION*: Cycloheximide is extremely toxic. Be sure to use proper personal protective equipment and hazardous waste disposal when handling solutions containing it.

*NOTE*: Experiments involving RNA require careful technique to prevent contamination.

*NOTE*: Use barrier tips where pipet tips are needed (e.g., 10‐µl barrier tips, Genesee Scientific, cat. no. 24‐401; 20‐µl barrier tips, Genesee Scientific, cat. no. 24‐404; 200‐µl barrier tips, Genesee Scientific, cat. no. 23‐412; and 1000‐µl barrier tips, Genesee Scientific, cat. no. 23‐430).

### Preparation

1 Put 1× PBS on ice.
2 Thaw cycloheximide and DTT on ice and add to NPB, CTIB, and HSWB (see recipes).
3 Keep NPB, CTIB, HSWB, and IB on ice.
4 Prepare an appropriate volume of NPB so it will ready immediately after tissue dissection (2 ml buffer per animal; see step 11).
5 Label five 1.7‐ml microcentrifuge tubes for each sample: one for homogenization, one for nuclei for INTACT, and three for ribosomal mRNA for TRAP (one to contain the protein G Dynabeads, one to contain anti‐GFP‐bound beads, and one to store the eluate from the RNA isolation).
6 Aliquot 1 ml NPB into each homogenization tube.
7 For each TRAP isolation, prepare one tube of washed protein G Dynabeads for pre‐clearing:
  - Gently resuspend beads by brief vortexing and pipet 50 µl beads into a 1.7‐ml microcentrifuge tube for each sample.
  - Place tube on a magnetic stand for 1 to 3 min.
  - Slowly pipet off buffer and add 500 µl NPB to each tube.
  - Gently resuspend by inversion and repeat steps 7b and 7c.
  - Place tube on the magnetic stand for 1 to 3 min and then slowly pipet off buffer.
  - Resuspend beads in 50 µl NPB and store on ice or at 4°C until ready for use.
8 For each TRAP isolation, prepare one tube of washed anti‐GFP‐coupled protein G Dynabeads for ribosomal GFP binding:
  - Gently resuspend beads by brief vortexing and pipet 100 µl of beads into a 1.7‐ml microcentrifuge tube for each sample.
  - Place tube on a magnetic stand for 1 to 3 min.
  - Slowly pipet off buffer and add 500 µl NPB to each tube.
  - Gently resuspend by inversion and repeat steps 8b and 8c.
  - Place tube on the magnetic stand for 1 to 3 min and slowly pipet off buffer.
  - Resuspend beads in 100 µl NPB.
  - Add 2 µl mouse anti‐GFP antibody.
  - Incubate for 1 hr at 4°C on a nutator.
  - Place tube on the magnetic stand for 1 to 3 min and pipet off buffer.
  - Add 200 µl NPB and gently resuspend by brief vortexing.
  - Place tube on the magnetic stand for 1 to 3 min, remove buffer, and resuspend in 100 µl NPB.
    Bead washes ensure that the solution that the beads and cells are suspended in is not affected by the buffer that the beads are shipped in.

### Tissue homogenization (Fig. 1, steps II and III, and Fig. 2, steps I and II)

9 Sacrifice *Emx1*‐NuTRAP mice (or another transgenic line expressing the NuTRAP cassette in a select cell type of interest) using methods approved by the Institutional Animal Care and Use Committee.
  We use rapid cervical dislocation at the age required for the experiment in order to capture gene expression unaltered by anesthetics or stress.
10 Dissect mouse brain to remove the whole hippocampal formation according to Sultan (2013):
  - Decapitate mouse using a razor blade.
  - Using gloved hands and forceps, peel away skin on the skull and use dissection scissors to open skull.
  - Remove brain and place on a sterile filter paper–covered petri dish (acting as a dissection stage). Rinse brain with cold 1× PBS.
  - Make a midsagittal cut using a razor blade.
  - Pin cerebellum with one spatula and, with another, peel back cortex.
  - With the spatula that pulled back the cortex, scoop out circumscribed curved hippocampal structure.
  - Transfer this hippocampus to another petri dish for the following steps.
    The tissue may be flash‐frozen on dry ice and stored ≤3 months at −80°C before use. You may use any other tissue as long as cells within the selected tissue express the NuTRAP cassette; however, this protocol is optimized for hippocampal tissue. To use this protocol with other tissues, you may have to adjust the volume of NPB used relative to the sample size. The goal would be to lyse the cells without disrupting the nuclear membranes or saturating the NPB solution with lipids (which would lead to an incomplete homogenization).
11 After tissue dissection, add about 700 to 800 µl NPB (from the 1‐ml aliquot from step 6) to tissue and use a razor blade to quickly chop and mince tissue.
  We have found that hippocampal tissue thawed after being flash‐frozen works well with this procedure.
12 Collect tissue using a P1000 pipet and add to the homogenization tube (total volume of 1 ml NPB).
13 Homogenize tissue on ice slowly and thoroughly by drawing through a 18G needle and 1‐ml syringe 50 times.
  Sometimes this will not fully homogenize the tissue, but we have found this amount of homogenization to be sufficient regardless. Experimenter judgment can be used here.

### Nuclear isolation (Fig 2, steps III, IV, and V2)

14 Add homogenized tissue sample to a 100‐μm cell strainer cap (yellow) on a 5‐ml tube.
  This helps separate the cells and more fully homogenize the sample.
15 Centrifuge 10 min at 1000 × *g*, 4°C, to filter supernatant. During this spin, prepare an appropriate volume of CTIB (200 µl per animal).
16 Wash SCD:
  - Resuspend beads by brief vortexing and add 100 µl SCD per animal to a 1.7‐ml microcentrifuge tube.
  - Incubate for 3 min on a magnetic stand.
  - Remove supernatant.
  - Add 1 ml NPB and vortex briefly.
  - Repeat steps 16b to 16d.
  - Resuspend beads in 100 µl NPB per 100 µl SCD used.
17 Resuspend cell pellet from step 15 (still in the 5‐ml tube) in the NPB that remains in the tube. Add sample to a 40‐µm cell strainer cap (blue) on a 5‐ml tube.
  This step aims to better lyse the cell membrane and leave only suspended nuclei in the solution.
18 Centrifuge 10 min at 1000 × *g*, 4°C, to filter supernatant.
19 Add 100 µl washed SCD (see step 16f) to each tube.
20 Resuspend beads by briefly pipetting up and down. Incubate for 20 min on ice and then resuspend and transfer resuspended beads to a 1.7‐ml microcentrifuge tube (INTACT tube).
21 Place tubes on a magnetic stand for 3 to 5 min to pull down the beads (nuclei are bound to these).
22 Slowly collect supernatant with a P1000 pipet and save for TRAP isolation procedure (see steps 27 to 35) in the ribosomal mRNA tube from step 5.
23 Slowly add 1 ml IB to beads while still on the magnetic stand to wash.
24 Pipet off IB slowly.
25 Repeat steps 23 and 24.
26 Ensure that beads do not dry out by continuing to a downstream analysis protocol immediately.
  At this step, the beads will be carrying the isolated nuclei. We use 100 µl IB to resuspend the nuclei and beads if we want to freeze for future downstream analysis. We use the Quick‐DNA Microprep Plus Kit (Zymo Research, cat. no. D4074; no modifications to the manufacturer's instructions) to isolate genomic DNA for quality control.

### TRAP isolation procedure (Fig. 2, steps V1 to VIII)

27 Add 100 µl CTIB to each 1 ml of supernatant from step 22.
  This brings the solution concentrations to that required for a normal TRAP isolation.
28 Centrifuge 10 min at 16,000 × *g*, 4°C, in a microcentrifuge.
29 Collect all clear supernatant and transfer to a tube containing 50 µl washed protein G Dynabeads from step 7f (avoid any pellet or top lipid layer).
  Optional: Save 50 µl of each sample in a new tube and keep on ice. RNA from this will be extracted for use as a total RNA input control.
30 Incubate for 30 min at 4°C on a nutator.
31 Incubate for 3 min at 4°C on a magnetic stand to pellet beads and transfer supernatant to a tube containing 100 µl washed protein G Dynabeads bound to anti‐GFP antibody from step 8k.
32 Incubate for 2 hr (or overnight) at 4°C on a nutator.
33 Slowly pipet off supernatant.
34 Wash beads three times by resuspending in 1 ml HSWB, incubating at 4°C on a magnetic stand for 3 min, and then removing supernatant.
35 Pipet off wash buffer and resuspend beads in 350 µl RLT buffer (from the RNeasy Mini Kit) by slowly pipetting up and down. Incubate for 30 min at 4°C on a nutator and then proceed to RNA purification.
  RLT buffer is necessary, as this contains reagent to bind RNA to the column.
  Steps 36 onward are the instructions from the RNeasy Mini Kit, with minor adaptations to apply to this protocol specifically.

### RNA purification

**Figure 3:**
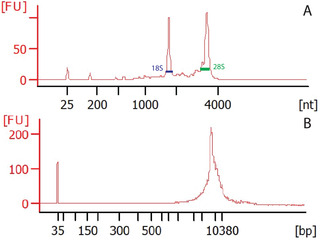
Representative Agilent Bioanalyzer traces for SIT‐isolated RNA (RNA Pico) (**A**) and DNA (high sensitivity) (**B**).

36 Incubate sample for 3 min at 4°C on a magnetic stand to pellet the beads. Meanwhile, prepare one new 1.7‐ml microcentrifuge tube as a collection tube for each TRAP‐isolated mRNA sample.
37 Add 350 µl of 70% ethanol to each tube.
38 Prepare one RNeasy spin column in a 2‐ml collection tube for each purification to be performed.
39 After the spin, carefully pipet the 350 µl supernatant out of each tube, leaving pelleted beads, and transfer to corresponding RNA tube containing the equal volume of 70% ethanol from step 37 (700 µl total volume per tube). Mix immediately by pipetting up and down.
40 To each RNA spin column, add the 700 µl ethanol/RNA mixture.
41 Centrifuge 15 s at 9500 × *g* and discard flowthrough (but save the collection tube).
42 Centrifuge 15 s at 9500 × *g* again and discard flowthrough.
43 Add 350 µl RW1 buffer to each column and centrifuge 15 s at 9500 × *g*. Discard flowthrough.
44 Prepare DNase solution by adding 10 µl stock RNase‐free DNase (at −20°C) to 70 µl RDD buffer (at 4°C) for each column. Mix by inverting and centrifuge briefly in a mini centrifuge.
45 Carefully add 80 µl DNase incubation mix directly to column matrix.
46 Incubate at room temperature for 15 min.
47 Add 350 µl RW1 buffer to each column and centrifuge 15 s at 9500 × *g*. Discard flowthrough.
48 Add 500 µl RPE buffer to each column and centrifuge 15 s at 9500 × *g*. Discard flowthrough.
49 Add 500 µl RPE buffer to each column and centrifuge 2 min at 9500 × *g*.
50 Carefully transfer column to a new 2‐ml collection tube, being sure not to let it contact the flowthrough to avoid ethanol contamination. Discard old collection tube and flowthrough.
51 Centrifuge 1 min at maximum speed.
52 Carefully transfer spin column to a 1.7‐ml collection tube from step 36.
53 Add 30 µl RNase‐free water directly to column matrix.
54 Centrifuge 1 min at 9500 × *g*.
55 Discard spin column, ensure that each tube is labeled correctly, and store at 80°C.
  This eluate contains the RNA.
56 Quantify RNA with a fluorometer and determine RNA quality using a bioanalyzer (see example data in Table 1 and Fig. 3).
  The RNA can now be used for downstream analysis.

**Table 1:** Qubit Concentrations

Type	Concentration (by Qubit)	RIN (if RNA)	Original tissue description	Sample
RNA	66.6 ng/μl	8.4	P42 mouse hippocampus	Unilateral
DNA	10.4 ng/ml		P42 mouse hippocampus	Unilateral

## REAGENTS AND SOLUTIONS

### Concentrated TRAP isolation buffer (CTIB)

- 10 µl 1 M HEPES, pH 7.5 (Fisher Scientific, cat. no. NC1584170)
- 117 µl 1 M MgCl_2_, molecular biology grade (Fisher Scientific, cat. no. AM9530G)
- 333 µl 3 M KCl, molecular biology grade (Alfa Aesar, cat. no. J64189, lot no. X18D048)
- 100 µl 100% IGEPAL (Sigma‐Aldrich, cat. no. I3021‐100ML)
- 1 µl 100 μg/µl cycloheximide, 98% (Fisher Scientific, cat. no. NC9651091)
- 275 µl 40 mg/ml sodium heparin (CAS no. 9041‐08‐1; Fisher Scientific, cat. no. BP2425)
- 20 µl 1 M DTT, molecular biology grade (Sigma‐Aldrich, cat. no. D9779‐1G)
- 110 µl 20 U/µl RNase inhibitor (Fisher Scientific, cat. no. N8080119)
- 20 µl 50× complete EDTA‐free protease inhibitor (Sigma‐Aldrich, cat. no. 11873580001; 1 tablet dissolved in 1 ml HyPure Water, Molecular Biology Grade, GE Life Sciences, cat. no. SH30538.03)
- 14 µl HyPure Water, Molecular Biology Grade (GE Life Sciences, cat. no. SH30538.03)
- Store ≤8 hr on ice (4°C)
- *We have also found that 10 µl 20 U/µl RNase inhibitor (Fisher Scientific, cat. no. N8080119) and 10 µl SUPERase Inhibitor (SUPERasin; Fisher Scientific, cat. no. AM2694) with 90 µl more HyPure Water, Molecular Biology Grade (GE Life Sciences, cat. no. SH30538.03) works well*.
- *Listed are volumes of reagents for 1 ml final volume. However, CTIB should be made fresh, with only the total volume needed prepared*.
- *Final concentrations: 10 mM HEPES, pH 7.5; 117 mM MgCl_2_; 1 M KCl; 10% IGEPAL CA‐630; 100 μg/ml cycloheximide; 11 mg/ml sodium heparin, 20 mM DTT; 2.2 U/μl RNase inhibitor; and 1× complete EDTA‐free protease inhibitor*.

### High‐salt wash buffer (HSWB)

- 250 µl 1 M Tris, pH 7.5 (from Tris base, molecular biology/proteomic grade, Genesee Scientific, cat. no. 18‐144)
- 600 µl 1 M MgCl_2_, molecular biology grade (Fisher Scientific, cat. no. AM9530G)
- 5 ml 3 M KCl, molecular biology grade (Alfa Aesar, cat. no. J64189, lot no. X18D048)
- 5 ml 10% IGEPAL CA‐630 (Sigma‐Aldrich, cat. no. I3021‐100ML)
- To 49.9 ml with HyPure Water, Molecular Biology Grade (GE Life Sciences, cat. no. SH30538.03)
- Store ≤1 week at 4°C
- Right before use, add 2 µl 1 M DTT, molecular biology grade (Sigma‐Aldrich, cat. no. D9779‐1G) per 1 ml buffer and 1 µl 100 μg/µl cycloheximide, 98% (Fisher Scientific, cat. no. NC9651091) per 1 ml buffer
- Once DTT and cycloheximide are added, store ≤8 hr on ice (4°C)
- *Final concentrations: 0.05 M Tris, pH 7.5; 0.012 M MgCl_2_; 0.3 M KCl; 1% IGEPAL CA‐630; 0.002 M DTT; and 100 µg/ml cycloheximide*.

### INTACT buffer (IB)

- 500 µl 1 M HEPES, pH 7.5 (Fisher Scientific, cat. no. NC1584170)
- 75 µl 1 M MgCl_2_, molecular biology grade (Fisher Scientific, cat. no. AM9530G)
- 167 µl 3 M KCl, molecular biology grade (Alfa Aesar, cat. no. J64189, lot no. X18D048)
- 4.275 g sucrose (Sigma‐Aldrich, cat. no. S1888‐500)
- 500 µl 10% IGEPAL (Sigma‐Aldrich, cat. no. I3021‐100ML)
- 1 ml 50× complete EDTA‐free protease inhibitor (Sigma‐Aldrich, cat. no. 11873580001; 1 tablet dissolved in 1 ml HyPure Water, Molecular Biology Grade, GE Life Sciences, cat. no. SH30538.03)
- To 50 ml with HyPure Water, Molecular Biology Grade (GE Life Sciences, cat. no. SH30538.03)
- Store ≤1 week at 4°C
- *Final concentrations: 10 mM HEPES, pH 7.5; 1.5 mM MgCl_2_; 10 mM KCl; 250 mM sucrose; and 0.1% IGEPAL CA‐630*.

### Nuclear preparation buffer (NPB)

- 500 µl 1 M HEPES, pH 7.5 (Fisher Scientific, cat. no. NC1584170)
- 75 µl 1 M MgCl_2_, molecular biology grade (Fisher Scientific, cat. no. AM9530G) or (Alfa Aesar, cat. no. J64189, lot no. X18D048)
- 167 µl 3 M KCl, molecular biology grade (Alfa Aesar, cat. no. J64189, lot no. X18D048)
- 4.275 g sucrose (Sigma‐Aldrich, cat. no. S1888‐500)
- 500 µl 10% IGEPAL (Sigma‐Aldrich, cat. no. I3021‐100ML)
- 1 ml 50× complete EDTA‐free protease inhibitor (Sigma‐Aldrich, cat. no. 11873580001; 1 tablet dissolved in 1 ml HyPure Water, Molecular Biology Grade, GE Life Sciences, cat. no. SH30538.03)
- To 49.8 ml with HyPure Water, Molecular Biology Grade (GE Life Sciences, cat. no. SH30538.03)
- Store ≤1 week at 4°C
- Right before use, add 0.2 µl 1 M DTT, molecular biology grade (Sigma‐Aldrich, cat. no. D9779‐1G) per 1 ml buffer and 1 µl 100 μg/µl cycloheximide, 98% (Fisher Scientific, cat. no. NC9651091) per 1 ml buffer
- Once DTT and cycloheximide are added, store ≤8 hr on ice (4°C)
- *Final concentrations: 10 mM HEPES, pH 7.5; 1.5 mM MgCl_2_; 10 mM KCl; 250 mM sucrose; 0.1% IGEPAL CA‐630; 0.2 mM DTT; and 100 μg/ml cycloheximide*.

## COMMENTARY

### Background Information

The *Emx1*‐NuTRAP mouse (Basic Protocol 1) was designed to answer questions about molecular changes in hippocampal excitatory neurons following environmental experiences, such as stress and exercise. The mouse expresses the NuTRAP cassette in *Emx1*‐expressing cells, which are primarily excitatory cortical neurons. We apply the approach of Roh and colleagues (Roh et al., 2017) to excitatory neurons in the hippocampus in order to discover transcriptional and epigenetic signatures underlying changes in neuronal function. This mouse allows for application of the SIT protocol (Basic Protocol 2) to nuclei and translating mRNA isolation as early as postnatal day 42 (P42) and likely sooner. Additionally, *Emx1*‐Cre labels neural progenitor cells, which may be disadvantageous for some but which has a unique advantage for those studying exercise or other experiences that impact neurogenesis. Increased neurogenesis, and therefore likely a change in neural progenitor cell function, has been demonstrated as key to the effect of exercise on memory (Marlatt, Potter, Lucassen, & van Praag, 2012).

SIT is a method for combining the previously established INTACT (Deal & Henikoff, 2011) and TRAP (Sanz et al., 2009) methods so that material from a single set of cells can be isolated together. The NuTRAP cassette contains a floxed STOP region, which allows for the labeling of any specific set of cells targeted by the selective expression of Cre recombinase (Roh et al., 2017). In the past, the Cre‐selective isolation of nuclei was typically done using INTACT, and mRNA was isolated with TRAP. These processes previously had incompatible buffers and were done on separate tissue isolates (Deal & Henikoff, 2011; Roh et al., 2017; Sanz et al., 2009). We developed a buffer set that preserves the necessary functionality of the TRAP buffers during the INTACT isolation while allowing the supernatant remaining from the INTACT method to be used in the TRAP method downstream. This was principally done by adding cycloheximide to the necessary INTACT buffers so that the ribosomes would be properly crosslinked to mRNA through the INTACT isolation. We demonstrated that this method produced isolated nuclei with DNA of sufficient quality for downstream applications (Fig. 3 and Table 1).

Our method's principal advantage over previous separate isolation methods is the direct comparison of multi‐omic sequencing data that can be made because it is from the same set of cells. In a tissue with as much heterogeneity as the brain, this becomes exceptionally important. Our process also allows for more material to be isolated from fewer samples. This could potentially permit use of fewer animals if the study design requires use of a specific region for each isolation. An advantage that our technique has over single‐cell sequencing is that our method preserves the sensitivity of bulk sequencing methods for low‐expression genes, which single‐cell sequencing loses (Lin et al., 2019). These low‐expression genes can be critical to determining the mechanism for changes in cell function. The potential disadvantage of our method is that it takes more time than individual isolation methods. However, given that we are able to isolate RNA with high RNA integrity numbers (RINs), the time that this method takes is not a significant concern. We have troubleshooted the method with frozen tissue, so concerns about maintenance of sufficient nuclear integrity for Basic Protocol 2 have also been mitigated.

We have used this method to compare transcriptomic data to epigenomic data from the same set of cells in an experiment to determine how voluntary aerobic exercise in early life might enable memory in adolescence (Raus et al., 2022). Fundamentally, using this transgenic mouse and this technique, we compared transcriptomic and epigenomic data from the same set of hippocampal excitatory neurons. Specific questions about other brain regions of excitatory neurons in the cortex could also be asked.

This technique could be applied to any tissue expressing the NuTRAP cassette. This would allow the researcher to ask questions at the intersection of epigenetics and translatomics in other cell or tissue types. The experimenter would need to optimize Basic Protocol 2 for each tissue used. Use in another tissue type would primarily require optimization of the ratio of buffers (especially the NPB buffer) to sample used. In rare cases, the concentration of detergent in the NPB buffer would need to be adjusted. This optimization of buffer volume or concentration ensures complete homogenization and cell membrane lysis without disruption of any nuclear membranes. Confirmation that the optimization was successful would require the same quality‐control analysis we describe here.

### Critical Parameters

#### Emx1‐NuTRAP mice (Basic Protocol 1)

It is critical that male breeders come from NuTRAP and not *Emx1*‐Cre mice. *Emx1* is sometimes active in sperm and could interfere with selective Cre expression.

#### SIT (Basic Protocol 2)

The most critical step is making sure the volume of NPB used is appropriate to the sample you are using. We find that 1 ml is sufficient to lyse the cell membranes of the bilateral P42 mouse hippocampus. The lipid content and volume of tissue used will heavily influence how much NPB is required. Insufficient NPB would lead to low yield and incomplete cell membrane lysis. Beyond that, there is little in this protocol open for modification; each step is critical to obtaining the results we report. The protocol should be performed as quickly as possible and on ice or in a refrigerator whenever possible. RNA can degrade quickly and degrades more quickly at room temperature. If some modification or tissue type requires longer incubation in the cycloheximide buffer, checking for excessive DNA damage by confirming that there are no small DNA fragments on a bioanalyzer may be appropriate.

### Troubleshooting

Please see Table 2 for a troubleshooting guide.

**Table 2:** Troubleshooting Guide for SIT

Problem	Possible cause	Solution
Low yield	Too much tissue used per volume of NPB buffer	Split the samples into smaller units and apply 1 ml NPB to each unit
Low mCherry signal in immunofluorescence around nucleus	Low mCherry expression	Amplify the signal with incubation with an antibody that binds an extra fluorophore

### Understanding Results

**Figure 4:**
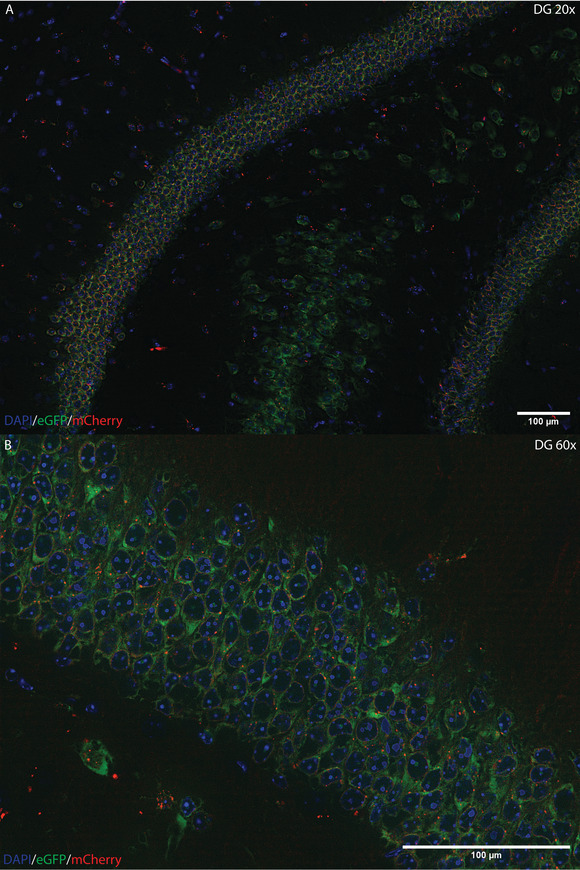
Dentate gyrus (DG) immunofluorescence images at 20× (**A**) and 60× (**B**) from 30‐µm hippocampal slices taken on the Keyence BZ‐X800 with use of the included haze‐reduction software.

The *Emx1*‐NuTRAP mouse (Basic Protocol 1) will express eGFP bound to ribosomes in hippocampal excitatory neurons and mCherry and biotin on the nuclear membranes of those neurons. On coronal brain slices, the eGFP and mCherry can be observed on excitatory pyramidal neurons in the cytoplasm and nuclear membrane, respectively. The eGFP should appear diffuse throughout the cytoplasm, and the mCherry should appear as puncta on the nuclear membrane. Both can be observed using a 20× objective lens (Fig. 4A) on an epifluorescent microscope but are best observed with a 60× oil‐immersion objective lens (Fig. 4B). As an example, we present images demonstrating the that the NuTRAP cassette is being selectively expressed in hippocampal granule cells, with potentially some inclusion of neural progenitor cells in the dentate gyrus (DG). DAPI is used to stain the nuclear material of all cells with a blue fluorescent signal. Lack of fluorescence in the granule cell layer would indicate that the NuTRAP cassette is not being expressed and that something went wrong with the cross. Low mCherry expression, or low signal, is normal. In Figure 4, we did not have to amplify the signal; however, signal amplification of the mCherry with a fluorescent antibody may be required to visualize the signal in some instances.

The SIT protocol (Basic Protocol 2) isolates ribosomes with eGFP and the mRNA bound to those ribosomes. We isolate the RNA with QIAGEN's RNeasy kit, as described in the protocol. We find the RNA to be highly pure and with high integrity (Fig. 3A) if the protocol is followed as described. The integrity can be determined by the RIN reported by the Agilent Bioanalyzer, which compares the ratio of the 28S peak to the 18S peak in the trace. The concentration can be determined using the bioanalyzer or using a Qubit fluorometer (Table 1). The concentration and integrity of the RNA that you need will be dependent on your downstream application. For RNA sequencing, we prefer to isolate ≥10 ng RNA with an RIN of at least 7, as recommended for use by PerkinElmer's NEXTFLEX Rapid RNA‐Seq Kit. To demonstrate our RNA quality, we show a representative bioanalyzer trace (Fig. 3A) along with the RIN as well as a table of Qubit‐reported concentrations in the supplemental information for any papers we publish. We also report the lowest RIN of all of the samples and state that all RINs were that or greater.

The SIT protocol also isolates biotin‐labeled nuclei. We use these nuclei directly in our downstream applications (ATAC‐seq and CUT&RUN‐seq). We occasionally visualize the nuclei before downstream applications to ensure they are intact (unpub. observ.). Here, we present a bioanalyzer trace (Fig. 3B) for DNA isolated from nuclei using Zymo Research's DNA Miniprep Kit (cat. no. D3024) for isolation of genomic DNA. Ensure that you do not detect sequences at a length that would interfere with your downstream application. Our trace has no or very little DNA below the peak at the base of the gel, indicating that most of the DNA remains intact or genomic (Fig. 3B). DNA shorter than 1000 bp would interfere with our downstream applications. It is also important to check the concentration of the isolated DNA to confirm that you have a sufficient quantity for your downstream application. We ensure the collection of ≥10 ng DNA for our applications. We determine the concentration using a Qubit fluorometer (Table 1) because the DNA will be genomic and not well analyzed by a bioanalyzer. Check your downstream protocol's requirements for integrity, quantity, and concentration. We recommend reporting your quality metrics with a representative bioanalyzer trace and a table of concentrations in your supplemental information. For a method that requires whole nuclei as an input, we recommend counting nuclei to determine the concentration of the nuclei isolated.

### Time Considerations

#### Emx1‐NuTRAP mice (Basic Protocol 1)

Mice breeding times can vary, but once born, *Emx1*‐NuTRAP mice can be used at any postnatal day for analysis, though we have only validated animals at P42 or later.

#### SIT (Basic Protocol 2)

For a team of two, this protocol requires ∼5.5 hr to go from eight live animals to the overnight TRAP incubation if all indicated preparation steps other than the ones that require solutions of fresh cycloheximide and DTT are taken care of the day before. Approximately 4 hr are required to complete the RNA isolation after the overnight TRAP incubation. For a team of two with eight animals, ∼11 hr are required to complete the protocol with no overnight TRAP incubation.

### Author Contributions

**Anthony M. Raus**: Conceptualization, Data curation, Formal analysis, Investigation, Methodology, Project administration, Supervision, Validation, Visualization, Writing – original draft, Writing – review and editing; **Nellie E. Nelson**: Formal analysis, Methodology, Validation, Writing – review and editing; **Tyson D. Fuller**: Conceptualization, Formal analysis, Investigation, Methodology; **Autumn S. Ivy**: Conceptualization, Funding acquisition, Methodology, Project administration, Resources, Supervision, Writing – review and editing.

### Conflict of Interest

The authors declare no conflict of interest.

## Data Availability

Please contact Autumn S. Ivy, MD, PhD, at aivy@uci.edu for data.
